# The English National Screening Programme for diabetic retinopathy 2003–2016

**DOI:** 10.1007/s00592-017-0974-1

**Published:** 2017-02-22

**Authors:** Peter H. Scanlon

**Affiliations:** 1 The English NHS Diabetic Eye Screening Programme, Gloucestershire Diabetic Retinopathy Research Group, Office above Oakley Ward, Cheltenham General Hospital, Sandford Road, Cheltenham, GL53 7AN UK; 2 Gloucestershire Hospitals NHS Foundation Trust, Cheltenham, UK

**Keywords:** Screening, Diabetic retinopathy, Blindness

## Abstract

The aim of the English NHS Diabetic Eye Screening Programme is to reduce the risk of sight loss amongst people with diabetes by the prompt identification and effective treatment if necessary of sight-threatening diabetic retinopathy, at the appropriate stage during the disease process. In order to achieve the delivery of evidence-based, population-based screening programmes, it was recognised that certain key components were required. It is necessary to identify the eligible population in order to deliver the programme to the maximum number of people with diabetes. The programme is delivered and supported by suitably trained, competent, and qualified, clinical and non-clinical staff who participate in recognised ongoing Continuous Professional Development and Quality Assurance schemes. There is an appropriate referral route for those with screen-positive disease for ophthalmology treatment and for assessment of the retinal status in those with poor-quality images. Appropriate assessment of control of their diabetes is also important in those who are screen positive. Audit and internal and external quality assurance schemes are embedded in the service. In England, two-field mydriatic digital photographic screening is offered annually to all people with diabetes aged 12 years and over. The programme commenced in 2003 and reached population coverage across the whole of England by 2008. Increasing uptake has been achieved and the current annual uptake of the programme in 2015–16 is 82.8% when 2.59 million people with diabetes were offered screening and 2.14 million were screened. The benefit of the programme is that, in England, diabetic retinopathy/maculopathy is no longer the leading cause of certifiable blindness in the working age group.

## Background

A reduction in diabetes-related blindness by at least one-third was declared a primary objective for Europe in 1989 in the St. Vincent Declaration [1]. Countrywide population-based diabetic retinopathy screening programmes have developed in Iceland (17,200 with diabetes [2] in 2015), Scotland (271,300 people [3] with diabetes), Wales (183,300 people [3] with diabetes), Northern Ireland (84,800 [3] people with diabetes) and England (2.91 million people [3] with diabetes). Regional and local screening programmes have developed in other parts of Europe [4] and around the world. The cost of the English Screening Programme is believed to be approximately 85.6 million US dollars or 40 US dollars per person screened.

The Wilson and Junger criteria for a screening programme, which are the 1968 principles [5] applied by the World Health Organisation, formed the basis of the UK National Screening Committee criteria for appraising the viability, effectiveness and appropriateness of a screening programme when the English NHS Diabetic Eye Screening Programme commenced in 2003. I previously described how we applied these principles to sight-threatening diabetic retinopathy to provide an evidence base [6, 7] for the development of the programme.

It is important to realise the following principles of screening:

1. Screening is a public health programme, not a diagnostic test.
2. Large numbers of apparently healthy individuals are invited for screening, and, if their screening test is positive, offer further diagnostic investigation.
3. Some people may be harmed by the process, or falsely reassured.
4. There is an ethical and moral responsibility to ensure that the programmes are of high quality.
5. Quality Assurance of Screening programmes is therefore essential to ensure that the programme achieves the highest possible standards and minimises harm.

These principles are fundamentally different to most branches of medicine where tests are considered to be diagnostic although, even in the circumstances of diagnostic tests, there will be some false positives and some false negatives.

The sensitivity of a screening test is the percentage of the condition that is correctly detected. If a screening test has a sensitivity of 90%, this means that 1 in 10 is missed. The specificity of a screening test is the percentage of people that one refers unnecessarily. If a screening test is 90% specific, this means 1 in 10 is referred unnecessarily. In 1995, a consensus view was put forward by clinicians at a meeting of the British Diabetic Association in Exeter that a screening test for diabetic retinopathy should have a minimum specificity of 80% and a specificity of 95%. Most studies on screening tests for diabetic retinopathy have achieved over 85% against a recognised reference standard and the specificity target of 95% has been achieved if the numbers with ungradable images are not calculated as test positive [8] but has proved more challenging to achieve when they have been calculated as test positive [9, 10].

It is important that any information that is sent to people who are offered screening tests explains to them that it will not detect all people with the disease and that a small number of people will be referred unnecessarily. It is also important to explain in the literature that a screening test for sight-threatening diabetic retinopathy will not pick up all other eye conditions.

## Stages in the development of the English NHS diabetic eye screening programme

When developing the NHS Diabetic Eye Screening Programme in England, we needed to consider 11 different stages that are listed in Table 1.

**Table 1:** Stages and considerations required in the development of the English Screening Programme

Stages	Considerations
1	Manoeuvring around the politics of funding
2	Are assessment and treatment facilities available?
3	Identify cohort for invitation and call—recall
4	How to invite them?
5	Informing the patients and maximising uptake
6	Establish an IT infrastructure
7	Choose a camera and decide on compression levels for photographs
8	The test
9	The grading referral criteria and viewing of the images
10	Employ and train a competent workforce
11	Introduce Quality Assurance

It was critical for diabetologists, ophthalmologists, public health doctors and optometrists to speak with one voice; otherwise, we would never have established the programme. Assessment and treatment facilities are available in England as part of our National Health Service, but this question becomes much more relevant in developing countries where treatment facilities may not be so readily available. There is no point in screening for sight-threatening diabetic retinopathy if treatment facilities are either not available or inadequate.

In England, everyone has a Primary Care Physician (GP) and so we have to obtain details from Primary Care on those diagnosed with diabetes and everyone has an NHS identifier number. A letter is sent out to everyone with diabetes aged 12 years and over to invite them for a diabetic eye screening appointment once a year. National leaflets have been produced to explain about diabetic retinopathy and about the screening test and what happens if screen-positive diabetic retinopathy is found. We have included information in the leaflet that the screening is not a diagnostic test and hence will only detect at best 90% of sight-threatening diabetic retinopathy and not other eye conditions. There has also been active engagement with patient organisations. There are appropriate exclusion criteria for those who do not need to be invited for screening, e.g. those already under ophthalmology and terminally ill.

Software has been developed to provide a single collated list of people with diabetes and for call recall, screening, grading and audit. In the database, the images are attached to patient details and confidentiality of patient data is a priority. The database is regularly backed up and an IT infrastructure has been established for capture and transmission of images to and from the cameras.

**Fig. 1:**
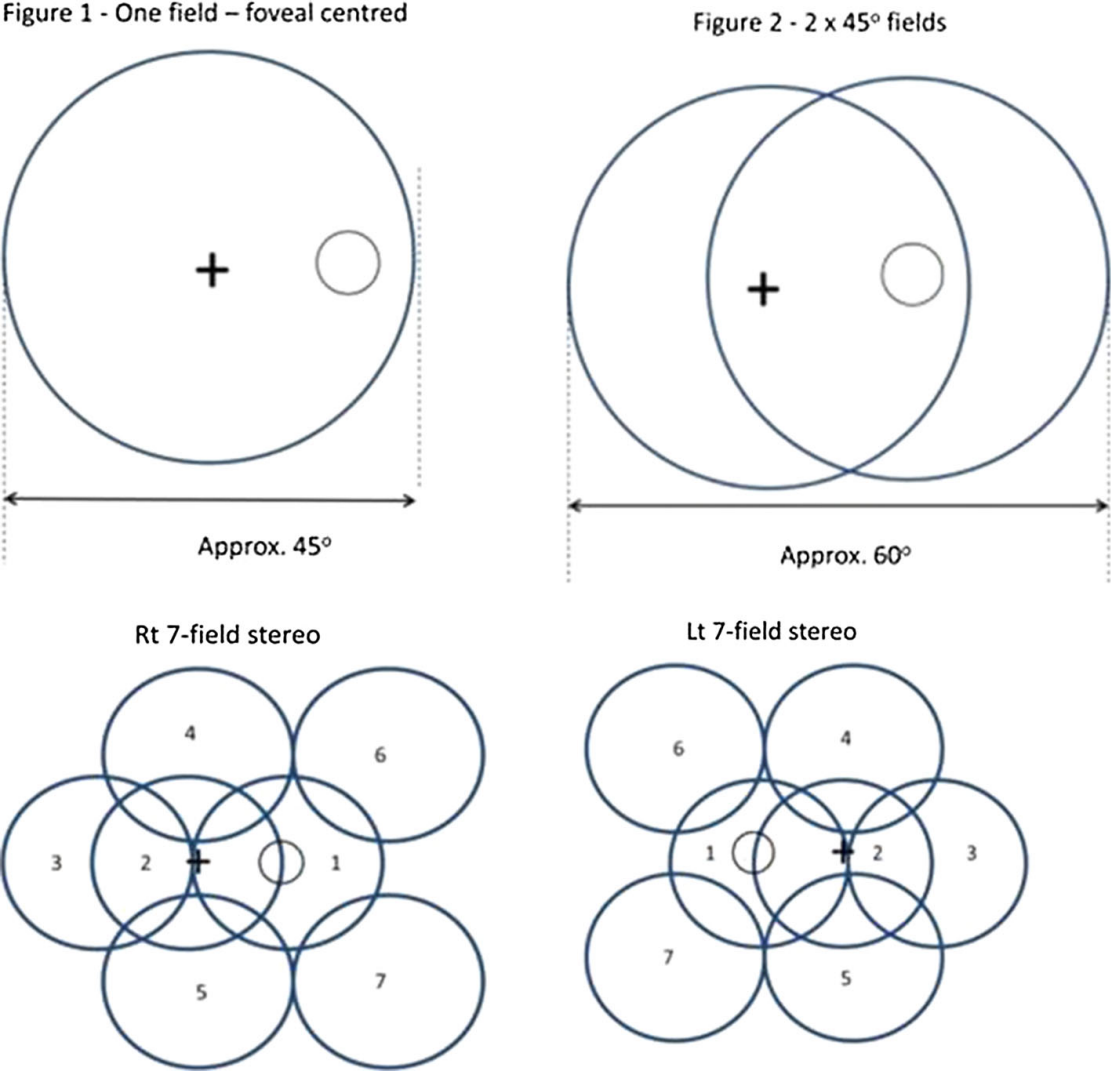
Photographic fields

In England, we use non-mydriatic cameras and undertake mydriatic photography on all people with diabetes aged 12 years and older. The two 45° fields captured by the English Screening Programme are shown in Fig. 1 together with the one 45° field used by Scotland and the seven 30° stereo fields that are used as a reference standard against which screening tests are judged. The English Screening Programme sets a minimum camera specification and tests all prospective cameras that meet this minimum specification on patients who are known to have specific features of diabetic retinopathy. The specification document is a fairly lengthy document which includes the following statements: The unit must be capable of providing a minimum field of view of 45° horizontally and 40° vertically at the specified resolution (at least 30 pixels/degree). The unit must be capable of accommodating refractive errors of ±15 D as detailed in EN ISO 10940. The internal fixation aid should be capable of positioning the eye to capture the fields of regard specified below. The ‘field of regard’ of the fundus camera must make it relatively straightforward for an appropriately trained and competent retinal screener to capture images centred on (1) the foveal area and (2) the optic disc. In addition, the ‘field of regard’ of the fundus camera must be able to capture images as defined by the area covered by fields 3–7 of the seven-field protocol used in the Early Treatment Diabetic Retinopathy Study [11]. The list of cameras that are currently approved to be used in the English Screening Programme is published by Public Health England on their webpage [12].

There has been a progressive increase in the size of uncompressed images from the modern camera backs which are now over 20 MB—the English NHS Diabetic Eye Screening Programme recommends capture of images that are compressed to a size of 1–2 MB. This level of compression has not been shown to lose any clinically significant information [13–15].

When considering whether to routinely dilate the pupil of people with diabetes attending for screening the study that was influential in the decision-making process was a population-based screening study [16] of 1549 people with diabetes who had received non-mydriatic one-field digital photography followed by mydriatic two-field digital photography and a reference standard examination by an experienced ophthalmologist whose examination was tested separately against seven-field stereo-photography [17]. The sensitivity for one-field non-mydriatic photography was 86.0% (95% CI, 80.9–91.1%), the specificity was 76.7% (95% CI, 74.5–78.9%) and the poor-quality image rate was 19.7% (95% CI, 18.4–21.0%). The sensitivity for two-field mydriatic photography was 87.8% (95% CI, 83.0–92.6%), the specificity was 86.1% (95% CI, 84.2–87.8%) and a poor-quality image rate was 3.7% (95% CI, 3.1–4.3%). This study led to the approach used in England of two-field mydriatic photography and the approach in Scotland of staged mydriasis with one-field non-mydriatic photography and with dilation only if poor-quality images were obtained. The correlation [18] with age led Northern Ireland to only routinely dilate those aged 50 years and over.

It has been demonstrated that there is a strong correlation [18] between age and poor-quality image rates in diabetic retinopathy screening, for both non-mydriatic and mydriatic photography. Hence, publications [19–22] with small numbers in a young age range are not relevant to population-based screening programmes where many of the individuals to be screened are over 60 years.

In any population-based screening programme, it is necessary to balance acceptability to the individuals being screened and cost-effectiveness of the screening method with detection rates of sight-threatening diabetic retinopathy. Population-based screening programmes that use non-mydriatic photography like the Scottish Screening Programme [23] usually capture one field centred on the fovea and those that use mydriatic photography like the English Screening Programme usually capture a second field that is centred on the disc, which also give a second view of the macular area. In 1989, Moss [11] demonstrated that for eight retinopathy levels, the rate of agreement with seven stereoscopic fields ranges from 80% for two 30° stereo fields to 91% for four 30° stereo fields. In 2003, Scanlon [17] reported that two-field mydriatic digital photography gave a sensitivity of 80.2% (75.2–85.2) and specificity of 96.2% (93.2–99.2) in comparison with seven-field stereo-photography. In the latter study, 15.3% of seven-field sets were ungradable compared with 1.5% of the two-field digital photographs.

Clear protocols need to be in place for management of people with poor-quality images. In the English Screening Programme, all people with poor-quality images are referred for examination by slit lamp biomicroscopy.

The English NHS Diabetic Eye Screening Programme routinely measures Visual Acuity at screening, but it is recognised that it is not sufficiently sensitive on its own to be a screening tool [24, 25]. Hence, it needs to be used in conjunction with other features that are detected at grading.

The diabetic retinopathy grading classification that has the best evidence base is the Early Treatment Diabetic Retinopathy Study (ETDRS) final Retinopathy Severity Scale [26] because it provided the first detailed classification system for retinopathy severity based on a natural history study of untreated eyes. However, this relies on detailed grading of stereo-photographs of seven fields of each eye. This scale did not grade lesions in the macular area. The ETDRS study did define ‘clinically significant macular oedema’ which was a level at which laser treatment was advised, but this was based on stereo-photography and the study did not recommend a referral level for closer observation before laser treatment was recommended.

Table 2 shows the International Classification [27] which was developed by the American Academy of Ophthalmology in 2002 and recommends that any level of retinopathy more severe than mild retinopathy (defined as the presence of microaneurysms only) warrants examination by an ophthalmologist. However, this is too early a referral level for use in the English Screening Programme and the referral level for the English Screening Programme is also listed in Table 2.

**Table 2:** International and English screening retinopathy classifications

‘International’ clinical classification [27]		English Screening Programme [48]
	Optimise medical therapy, screen at least annually	R0 Currently screen Annually
Ma’s only		R1 Screen annually Background Microaneurysm(s) or HMa Retinal haemorrhage(s) Venous loop Any exudate or cotton wool spots (CWS) in the presence of other non-referable features of DR
More than just micro aneurysms but less severe than severe NPDR	Refer to ophthalmologist	
		R2 Refer to ophthalmologist Pre-proliferative Venous beading Venous reduplication Intraretinal microvascular abnormality (IRMA) Multiple deep, round or blot haemorrhages
Severe NPDR Any of the following: (a) Extensive intraretinal haem (>20) in 4 quadrants (b) Definite venous beading in 2+ quadrants (c) Prominent IRMA in 1+ quadrant And no signs of PDR	Consider Scatter photocoagulation for type 2 diabetes	
Neovascularisation Vitreous/pre-retinal haemorrhage	Scatter Photocoagulation without delay for patients with vitreous haemorrhage or neovascularisation within 1 disc diameter of the optic nerve head	R3A Urgent referral to ophthalmologist R3A. Proliferative New vessels on disc (NVD) New vessels elsewhere (NVE) Pre-retinal or vitreous haemorrhage Pre-retinal fibrosis ± tractional retinal detachment
		R3S Follow-up annually within screening or at appropriate interval in surveillance R3S. Stable treated proliferative Evidence of peripheral retinal laser treatment AND Stable retina from photograph taken at or shortly after discharge from the hospital eye service (HES)

Table 3 shows the risks of progression to proliferative diabetic retinopathy as recorded in the Early Treatment Diabetic Retinopathy Study [26]. Screening Programmes need to accept a certain level of risk. In the English programme, we needed to decide whether we were prepared to accept a 6.2% risk or an 11.3% risk that a patient who has been screened and given a 1-year appointment develops proliferative DR before their next screen. We opted for the 11.3% risk which is the equivalent to moderate non-proliferative diabetic retinopathy on the ETDRS final Retinopathy Severity Scale [26]. We also had to develop a definition for maculopathy referral based on two-dimensional markers. The ETDRS study did not classify maculopathy, but it did make recommendations on what constituted clinically significant macular oedema requiring laser treatment as shown in Table 4. We opted for three referral criteria, based on two-dimensional photographic markers and measurement of Visual Acuity:

**Table 3:** ETDRS Diabetic Retinopathy Classification of Progression to Proliferative DR

ETDRS final retinopathy severity scale [26]	ETDRS (final) grade	Lesions	Risk of progression to PDR in 1 year (ETDRS interim)
No apparent retinopathy	10 14, 15	DR absent DR questionable	
Mild non-proliferative diabetic retinopathy (NPDR)	20	Micro aneurysms only	
	35 a b c d e	One or more of the following: Venous loops > definite in 1 field SE, IRMA, or VB questionable Retinal haemorrhages present HE > definite in 1 field SE > definite in 1 field	Level 30 = 6.2%
Moderate NPDR	43a b	H/Ma moderate in 4–5 fields or severe in 1 field or IRMA definite in 1–3 fields	Level 41 = 11.3%
Moderately severe NPDR	47 a b c d	Both level 43 characteristics – H/Ma moderate in 4–5 fields or severe in 1 field and IRMA definite in 1–3 fields **or** any one of the following: IRMA in 4–5 fields HMA severe in 2–3 fields VB definite in 1 field	Level 45 = 20.7%
Severe NPDR	53 a b c d	One or more of the following: > 2 of the 3 levels, 47 characteristics H/Ma severe in 4–5 fields IRMA > moderate in 1 field VB > definite in 2–3 fields	Level 51 = 44.2% Level 55 = 54.8%
Mild PDR	61a b	FPD or FPE present with NVD absent or NVE = definite	
Moderate PDR	65a b	(1) NVE > moderate in 1 field or definite NVD with VH and PRH absent or questionable or (2) VH or PRH definite and NVE < moderate in 1 field and NVD absent	
High-risk PDR	71 a b c d	Any of the following: (1) VH or PRH > moderate in 1 field (2) NVE > moderate in 1 field and VH or PRH definite in 1 field (3) NVD = 2 and VH or PRH definite in 1 field (4) NVD > moderate	
High-risk PDR	75	NVD > moderate and definite VH or PRH	
Advanced PDR	81	Retina obscured due to VH or PRH

**Table 4:** ETDRS Maculopathy Classification

Early treatment diabetic retinopathy study	Outcome
Clinically significant macular oedema [49] as defined by	
A zone or zones of retinal thickening one disc area or larger, any part of which is within one disc diameter of the centre of the macula	Consider laser
Retinal thickening at or within 500 microns of the centre of the macula	Consider laser
Hard exudates at or within 500 microns of the centre of the macula, if associated with thickening of the adjacent retina (not residual hard exudates remaining after disappearance of retinal thickening)	Consider laser

1. Exudate within 1 disc diameter (DD) of the centre of the fovea (Fig. 2).

**Fig. 2:**
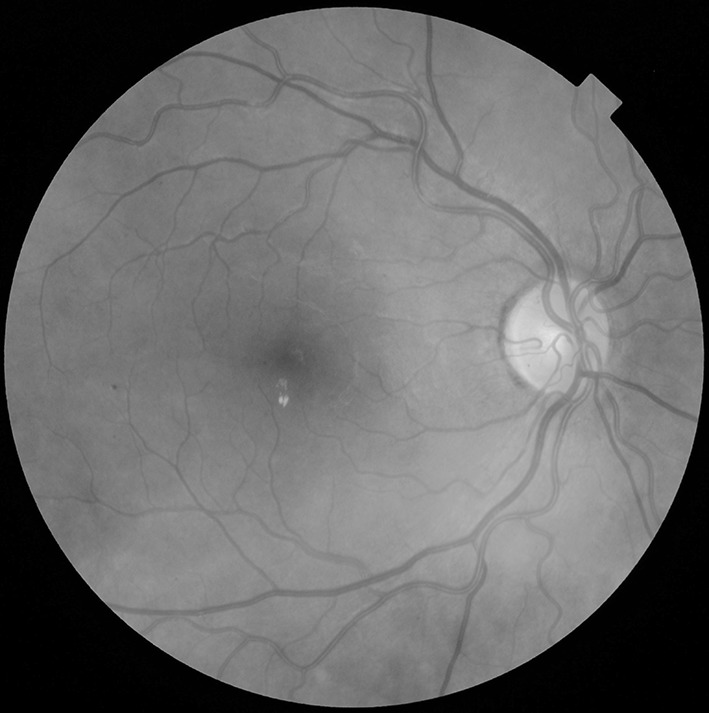
Exudate within 1 disc diameter (DD) of the centre of the fovea
2. Circinate or group of exudates within the macula (Fig. 3).

**Fig. 3:**
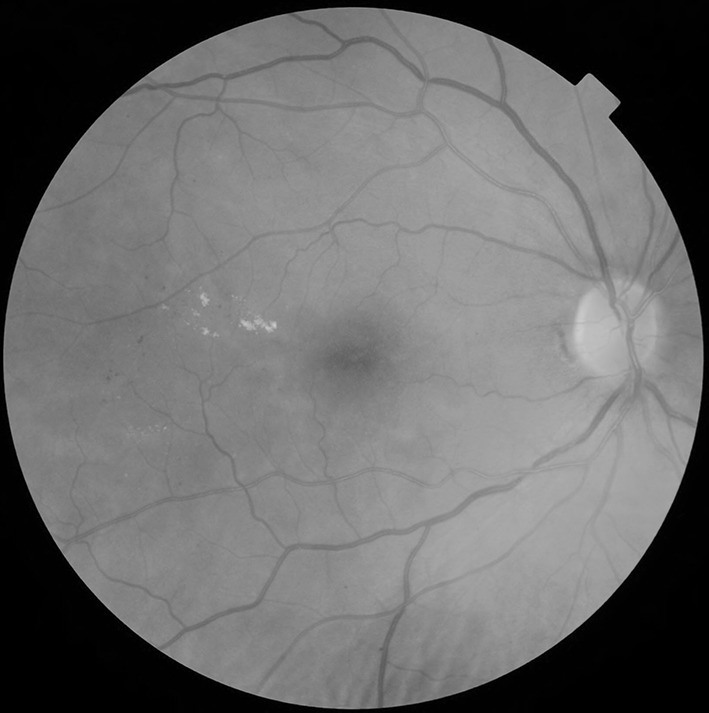
Circinate or group of exudates within the macula
3. Any microaneurysm or haemorrhage within 1DD of the centre of the fovea only if associated with a best VA of ≤ 6/12 (if no stereo) (Fig. 4).

**Fig. 4:**
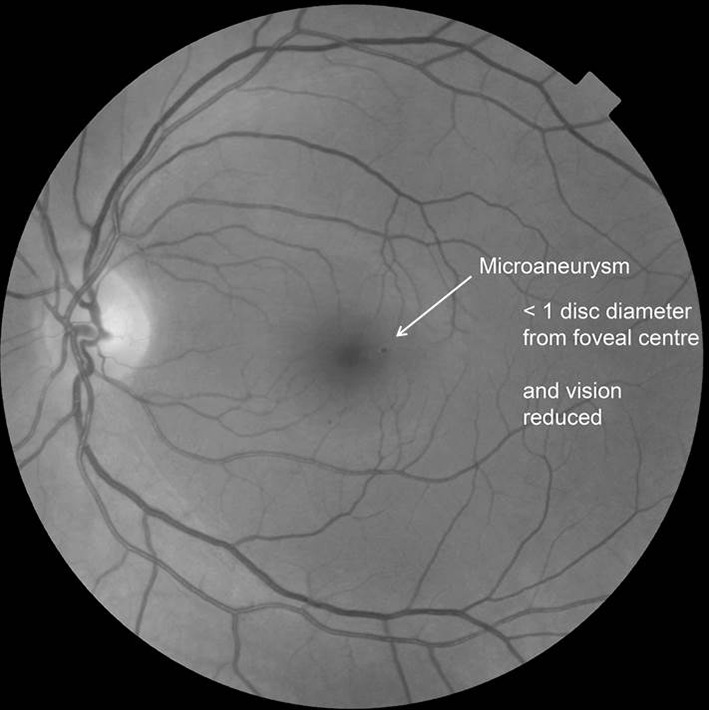
A microaneurysm within 1DD of the centre of the fovea associated with a best VA of ≤ 6/12

A minimum screen resolution is recommended [12] when viewing the images for grading which has progressed as the technology of screens has advanced. The current minimum acceptable standard for screen resolution is a vertical resolution of 1080 (1920 × 1080) with an achievable and recommended standard of a minimum of 1200 (1920 × 1200 or higher). It is recommended that a minimum of 60% of the image should be viewable on the grading screen to avoid too much scrolling to see the full image.

To ensure that whole screening programme is provided by a trained and competent workforce a minimum qualification [28] is required for screeners and graders in the English programme. Evidence of ongoing continuous professional development and taking the monthly External Quality Assurance Test sets [29, 30] is also required—all 1500 graders in the English Screening Programme are required to take a monthly test set of 20 image sets and their grading of these images is compared against a guide grade. An International Version of the qualification [31] and the monthly test and training set [32] for screeners working outside the UK is available.

An important part of any screening programme is the introduction of Quality Assurance. The purpose of introducing Quality Assurance is to reduce the probability of error and risk, ensure that errors are dealt with competently and sensitively, help professionals and organisations improve year on year, and set and keep under review national standards.

The NHS Diabetic Eye Screening Programme has developed three Key Performance Indicators and nine other Quality Standards [33]. These are given in Table 5 with the three Key Performance Indicators being shown in the right-hand column. A programme board which includes local health service representatives and national Quality Assurance team representatives oversees the results of a programmes performance against the standards four times a year, and if a programme is performing poorly, they are expected to improve or the service may be recommissioned to be provided by a different provider. Graders who perform poorly on test sets undergo extra training and have all of their work second graded until their performance improves.

**Table 5:** Standards and key performance indicators in the English NHS Diabetic Eye Screening Programme

Standard	Criteria	Thresholds	Key performance indicators
1	Proportion of the known eligible people with diabetes offered an appointment for routine digital screening	Acceptable: ≥ 95% Achievable: ≥ 98%	
2	Proportion of people newly diagnosed with diabetes offered a first routine digital screening appointment that is due to occur within 89 calendar days of the programme being notified of their diagnosis	Acceptable: ≥ 90% Achievable: ≥ 95%	
3	Proportion of eligible people with diabetes offered an appointment for routine digital screening occurring 6 weeks before or after their due date	Acceptable: ≥ 95% Achievable: ≥ 98%	
4	Proportion of people with diabetes offered an appointment for slit lamp biomicroscopy 6 weeks before or after their due date	Thresholds, to be set	
5	Proportion of people with diabetes on digital surveillance who have been offered an appointment that occurs within a reasonable time of their follow-up period	Thresholds, to be set	
6	Proportion of pregnant women with diabetes seen within 6 weeks of notification of their pregnancy to the screening programme	Thresholds, to be set	
7	The proportion of those offered routine digital screening who attend a digital screening event where images are captured	Acceptable: ≥ 75% Achievable: ≥ 85%	KPI 1
8	Proportion of eligible people with diabetes who have not attended for screening in the previous 3 years	Thresholds, to be set	
9	Proportion of eligible people with diabetes where a digital image has been obtained but the final grading outcome is ungradable	Acceptable: 2–4%	
10	Time between routine digital screening event or digital surveillance event or slit lamp biomicroscopy event and printing of results letters to the person with diabetes, GP and relevant health professionals	Acceptable: 85% < 3 weeks and 99% < 6 weeks.	KPI 2
11	Time between routine digital screening event or digital surveillance event or slit lamp biomicroscopy event and issuing the referral to the hospital eye service	1. Urgent Acceptable: ≥ 95% 2 weeks Achievable: ≥ 98% 2 weeks 2. Routine Acceptable: ≥ 90% 3 weeks Achievable: ≥ 95% 3 weeks	
12	Time between screening event and first attended consultation at hospital eye services or digital surveillance	1. Urgent Acceptable: ≥ 80% 6 weeks Achievable: ≥ 95% 6 weeks 2. Routine Acceptable: ≥ 70% 13 weeks Achievable: ≥ 95% 13 weeks	KPI 3
13	Time between digital screening event and first attended consultation in slit lamp biomicroscopy surveillance	Acceptable: ≥ 70% within 13 weeks Achievable: ≥ 95% within 13 weeks	

In addition, an External Quality Assurance visit to all regional programmes who undertake Diabetic Eye Screening as part of the NHS Diabetic Eye Screening Programme is undertaken every 3 years. EQA visits are an integral part of Diabetic Eye Screening Quality Assurance. Formal EQA visits to a screening programme provide the forum for a review of the whole multidisciplinary screening pathway and an assessment of the effectiveness of team working within the screening centre and associated referral sites.

## Programme results

In the development of the programme, I calculated [7] that the NHS Diabetic Eye Screening Programme had the potential to reduce the prevalence of blindness in England from 4200 people to under 1000 people based on UK certification of blindness. If WHO definitions were used the prevalence, incidence and potential reductions in blindness are much greater. In 2014, Liew [34] reported on the causes of blindness certifications in England and Wales in working age adults (16–64 years) in 2009–2010 and compared these with figures from 1999 to 2000. For the first time in at least five decades, diabetic retinopathy/maculopathy was no longer the leading cause of certifiable blindness amongst working age adults in England and Wales, having been overtaken by inherited retinal disorders. This change was considered to be due to the introduction of nationwide diabetic retinopathy screening programmes in England and Wales and improved glycaemic control. The era in which this reduction in blindness occurred was during the period when laser treatment was being used for maculopathy and before the use of VEGF inhibitors for diabetic macular oedema.

In 2015–2016, the NHS Diabetic Eye Screening Programme in England [35] offered screening to 2,590,082 with diabetes using two-field mydriatic digital photography. There were 3,083,401 known people with diabetes in England, but people who are under an ophthalmologist for diabetic eye disease and certain other categories of people (e.g. terminally ill) are not invited. A total of 2,144,007 with diabetes were screened (Uptake 82.8%). New registrations to programmes in 2015–2016 were 326,587. There were 7593 urgent referrals with proliferative retinopathy and 52,597 referrals with screen-positive maculopathy or pre-proliferative diabetic retinopathy. Rate of retinopathy per 100,000 screened was 2807.

### Future developments for the programme

Changes in technology have introduced three-dimensional imaging in the form of Optical Coherence Tomography. These machines are more costly than digital cameras and are not felt to be cost-effective as a first-line screening tool when 65% of the population of people with diabetes have no retinopathy. However, there is a high possibility that they will be introduced as a second-line screening tool for screen-positive maculopathy using two-dimensional markers. It is believed that, of the 52,597 referrals with screen-positive maculopathy, only 20% actually require treatment and a significant proportion of the remaining 80% could be followed up in a technician-led clinic [36] that includes OCT images to exclude any significant diabetic macular oedema. Cost-effectiveness data are needed before this can be introduced.

Extensive work has been done in the area [37–40] of extended screening intervals for those at low risk. The UK National Screening Committee agreed at their committee on 19 November 2015 and published their recommendation in January 2016 that:

- For people with diabetes at low risk of sight loss, the interval between screening tests should change from 1 to 2 years.
- The current 1 -year interval should remain unchanged for the remaining people at high risk of sight loss.

The introduction of this extension of screening interval for those with no retinopathy on two consecutive screens, which is the current recommendation in England, is dependent on software development for the programme.

The use of automated analysis is currently being evaluated for use in the English Screening Programme. A recent HTA report [41] has been published on this topic. There are different ways in which automated analysis could be used:

- To classify images as no diabetic retinopathy or diabetic retinopathy so that a human grader would only need to look at those with diabetic retinopathy.
- To detect referral levels of retinopathy.
- To act as a quality assurance tool for retinopathy that is missed.
- To determine which images are gradable and which are ungradable.

Scanning laser ophthalmoscopes and wide-field imaging have been widely studied [42–44], but this method has not yet been shown to be cost-effective. The earlier devices that provided wide-field imaging compromised [45] on the detection of microaneurysms in the central field.

No hand-held device has ever been shown [46] to have comparable sensitivities and specificities for the detection of sight-threatening diabetic retinopathy to devices where the camera is fixed and the patient’s head is placed on a chin rest and forehead against a fixed band and cannot, therefore, be recommended for population-based screening at the present time.

OCT angiography is new technology [47] that is not currently suitable for population-based screening.

## Conclusions

Screening for sight-threatening diabetic retinopathy has been shown to be very effective in England in reducing blindness due to diabetic retinopathy and reducing the number of vitrectomies being performed on advanced disease.
